# Study of the Ability of Gauze to Filter Bacterial Spray from the Air with Special Reference to Mouth Spray

**Published:** 1919

**Authors:** 


***Study of the Ability of Gauze to Filter Bacterial Spray from the Air with Special Reference to Mouth Spray.*** Abstract of Report from Dr. George H. Weaver. Received October 4, 1918.

The first tests were made with a spray of carbol-fuchsin and one of bacterial suspension. In these it was demonstrated that gauze will remove bacteria from the air. The efficiency of the gauze as a filter is in direct ratio to the fineness of the mesh and the number of layers used.

Experiments with a mouth spray were taken up. "A suitable subject for these tests was found in an adult who was the subject of a chronic antrum and ethmoid suppuration with constant purulent discharge in the throat and mouth; abundant streptococcus viridans was constantly present. It has been noted by those who have studied the bacteria of mouth sprays that the number of bacteria discharged is exceedingly variable when coughing efforts are made. We found that when our subject coughed with mouth wide open few bacteria were driven out, but that an explosive cough with the lips held quite close yielded quite a rich bacterial spray. An abundant bacterial spray was obtained by first distending the cheeks with air and then instantly opening the lips and immediately forcing the air out with a puff. The tests were made by having the subject direct such forcible expiratory efforts towards Petri dishes containing blood agar, at a distance of six inches, the test dishes being uncovered and covered by various gauzes in different multiples."

The result of many tests was to show that there is no appreciable difference between dry and moist gauze in filtering properties. Three or four layers of gauze with a measure of 44 × 40 was found to remove most of the bacterial spray of short distances. The masks, however, were not entirely bacteria-tight, and it was found that at considerable distances a larger proportion of the bacteria reaching the gauze passed through, probably because only very small droplets are carried such distances.

The Durand hospital has adopted a mask of three layers of gauze 44 × 40 for ordinary use, and in all circumstances when the danger from mouth spray is great two such masks are super-imposed. The most favorable type are (*a*) oblong, with the tapes at each corner; and (*b*) a pattern with only two tapes sewed to the gathered ends of the mask.

